# Brief Report: Translating Selection Criteria for Lung Cancer Screening to an Indian Context—An Analysis From a Tertiary Health Centre

**DOI:** 10.1016/j.jtocrr.2026.100987

**Published:** 2026-03-16

**Authors:** Thomas Callender, Amyn Bhamani, Sneha Verma, Ayush Goel, Tejas Suri, John R. Hurst, Neal Navani, Anant Mohan

**Affiliations:** a Department of Public Health and Primary Care, University of Cambridge, Cambridge, United Kingdom; b Lungs for Living Research Centre, University College London (UCL) Respiratory, University College London, London, United Kingdom; c Department of Pulmonary, Critical Care, and Sleep Medicine, All India Institute of Medical Sciences, New Delhi, India; d UCL Respiratory, University College London, London, United Kingdom; e University College London Hospitals National Health Service (NHS) Foundation Trust, London, United Kingdom

**Keywords:** Lung neoplasms, Early detection of cancer, Epidemiology, Risk assessment, Smoking, Healthcare disparities, India

## Abstract

**Background:**

Lung cancer is the most common cause of cancer in India, leading to growing interest in the potential for screening. Established lung cancer screening programs exist in both the U.K. and U.S. contexts, using either combinations of risk factors (United States) or risk models (United Kingdom) to determine eligibility. Whether these approaches can be directly translated to an Indian context to determine eligibility for lung cancer remains unknown.

**Methods:**

We analyzed a case series of individuals newly diagnosed or under active follow-up for lung cancer at a large tertiary referral center in northern India. We considered eligibility for lung cancer on the basis of the US Preventive Services Taskforce 2021 criteria, and predicted risk using the lung cancer incidence (UCL-I), the lung cancer discrimination (UCL-D), the Prostate, Lung, Colorectal, and Ovarian Cancer Screening Trial modified 2012 lung cancer risk prediction (PLCOm2012), and Liverpool Lung Project (LLP) models.

**Results:**

A total of 472 people with lung cancer were included. The median age at diagnosis of lung cancer was 60 years, and 83.7% of the cohort were men. Most were never- (30.5%) or former (47.3%) smokers. 97% of lung cancers were diagnosed at stages III or IV. Nearly half of the individuals were under the age of 55 or were never-smokers and so would not have been considered for risk assessment under U.K. screening criteria. Fewer than 40% of ever-smokers would have met thresholds for lung cancer screening used in the United States or the United Kingdom.

**Conclusion:**

Criteria used to determine eligibility for lung cancer screening in the U.K. and U.S. settings are unlikely to be translatable to an Indian context without modification. Prospective screening studies should be considered before widespread implementation in India.

## Introduction

Lung cancer is one of the most common causes of cancer in India, with pronounced regional differences and an urban/rural split in incidence rates.^1^ Risk-stratified lung cancer screening has been found to reduce deaths from lung cancer by at least 20% among those screened and has been recommended in several high-income countries, including the United Kingdom and the United States.^2^, ^3^, ^4^, ^5^ This has led to increased discussion of the potential of screening within an Indian context.^6^

By limiting screening to those at higher risk of lung cancer—on the basis of either a combination of age and smoking history or explicitly estimated by a risk model—existing lung cancer programs in high-income countries have been designed to maximize their benefit/harm profile and cost-effectiveness. However, to our knowledge, there have been no previous attempts to quantify the risk of lung cancer in Indian populations using existing risk scores.^7^

Large-scale hospital case series and national registry data have highlighted the variation in age-standardized rates of lung cancer across the regions of India, along with the younger mean age at presentation.^1^^,^^8^^,^^9^ Furthermore, patterns of smoking differ within an Indian context, with nearly three-quarters smoking bidis (thin, hand-rolled cigarettes).^10^ Correspondingly, it is unlikely that existing screening criteria in use in high-income countries will perform equally well within different geographic and socio-cultural settings. Using a large case series from an urban tertiary hospital, we sought to characterize the risk factors of those diagnosed with lung cancer to assess patterns of risk factors amongst this cohort and the degree to which existing lung cancer screening eligibility criteria might be translated to an Indian context.

## Methods

Data were collected from August 2024 to April 2025 from individuals newly diagnosed with lung cancer or under active treatment or follow-up in the pulmonary department of a tertiary health care center in New Delhi, India. From each participant, we collected demographic, socioeconomic, smoking, medical history, and family history information including age, sex, ethnicity, education level, socioeconomic status, history of asbestos exposure, smoking history, biomass fuel exposure, comorbidities, and family history of malignancy. Cumulative biomass exposure from domestic cooking was quantified as the product of the number of hours per day and the number of years of such exposure.

We analyzed the cohort using standard descriptive statistics, in aggregate, and for current, former, and never-smokers (defined as those who have smoked fewer than 100 cigarettes or bidis in their lifetime) separately. Then, we assessed predicted risk of lung cancer among ever-smokers using the lung cancer incidence mode (UCL-I)^11^ (5-y risk of lung cancer incidence ≥1.17%), lung cancer discrimination model (UCL-D) (5-y risk of lung cancer death of ≥0.68%), the Prostate, Lung, Colorectal, and Ovarian Cancer Screening Trial modified 2012 lung cancer risk prediction (PLCOm2012) model^12^ (6-y risk of lung cancer incidence of ≥1.51%), Liverpool Lung Project (LLP) versions 2 and 3^13^(5-y risk of lung cancer incidence of ≥2.5%) models, and the US Preventive Services Taskforce (USPSTF) 2021 criteria.^4^ The USPSTF recommends lung cancer screening on the basis of a combination of risk factors, including those aged 50 to 80 years who have accumulated at least 20 pack-years of smoking exposure and, if a former smoker, have quit within the past 15 years.^4^

As a case series at the point of lung cancer diagnosis, this study could not assess the performance metrics of any predictive model. Instead, we provide an estimate of the proportion of individuals who might have been eligible for lung cancer screening, given existing risk models and thresholds for screening and the characteristics of those eligible. This will inform the design of future studies regarding risk modeling and lung cancer screening in an Indian context.

This study was approved by the All-India Institute of Medical Sciences (AIIMS) Ethics Committee, reference: AIIMS A1036/05.03.2024, RP-13/2024, with all participants providing informed consent.

## Results

We analyzed data on 472 individuals with lung cancer incidence. Individuals were comparatively young, with a median age of 60 years (interquartile range: 53–68), 83.7% were men, 57.2% had a primary school level of education or less, and 20.6% were farmers (Table 1). Just under one-third of the cohort were reported to be never-smokers (n = 144, 30.5%), and half (n = 223, 47.3%) were former smokers. Among ever-smokers, 82.1% exclusively reported smoking bidis, 8.8% cigarettes, and 9.1% both bidis and cigarettes, with more current smokers smoking only bidis compared with former smokers (87.6% versus 79.4%, respectively). A plurality of the cohort was diagnosed at stage IV (64.0%), with only 3.0% diagnosed at either stage I (n = 3) or II (n = 11). The common histologic diagnosis was adenocarcinoma (n = 184, 39.1%) followed by squamous cell carcinoma (n = 100, 21.2%) (Supplementary Tables 1 and 2).

**Table 1:** Descriptive Characteristics Stratified by Smoking Status

Variable	Full Cohort n = 472	Current Smokers n = 105	Former Smokers n = 223	Never-Smokers n = 144
Age (mean, SD) y	59.7 (10.6)	60.8 (8.5)	61.7 (9.7)	55.6 (12.1)
Sex - Female (n, %)	77 (16.31%)	2 (1.90%)	18 (8.07%)	57 (39.58%)
BMI (mean, SD) kg/m^2^	21.8 (4.0)	21.2 (3.6)	21.4 (3.7)	22.8 (4.4)
Education (n, %)				
Illiterate	139 (29.51%)	18 (17.14%)	68 (30.63%)	53 (36.81%)
Primary	131 (27.81%)	44 (41.90%)	66 (29.73%)	21 (14.58%)
Secondary (Matric)	88 (18.68%)	23 (21.90%)	36 (16.22%)	29 (20.14%)
Higher secondary (intermediate)	52 (11.04%)	10 (9.52%)	31 (13.96%)	11 (7.64%)
Graduate	47 (9.98%)	7 (6.67%)	17 (7.66%)	23 (15.97%)
Postgraduate	14 (2.97%)	3 (2.86%)	4 (1.80%)	7 (4.86%)
Occupation (n, %)				
Farming	97 (20.55%)	19 (18.10%)	68 (30.49%)	10 (6.94%)
Laborer/skilled worker	178 (37.71%)	60 (57.14%)	90 (40.36%)	28 (19.44%)
White collar/office job	75 (15.89%)	14 (13.33%)	36 (16.14%)	25 (17.36%)
Unemployed	2 (0.42%)	0 (0.00%)	2 (0.90%)	0 (0.00%)
Retired/housewife	116 (24.58%)	11 (10.48%)	24 (10.76%)	81 (56.25%)
Other	4 (0.85%)	1 (0.95%)	3 (1.35%)	0 (0.00%)
Biomass exposure (n, %)	138 (29.24%)	12 (11.43%)	54 (24.22%)	72 (50.00%)
Asbestos exposure (n, %)	55 (11.65%)	17 (16.19%)	30 (13.45%)	8 (5.56%)
COPD^a^ (n, %)	87 (18.43%)	33 (31.43%)	45 (20.18%)	9 (6.25%)
Tuberculosis (n, %)	83 (17.58%)	19 (18.10%)	40 (17.94%)	24 (16.67%)

Note: All data were complete except for education and asbestos exposure, in which one former smoking individual had no data.

BMI, body mass index; COPD, chronic obstructive pulmonary disease.

a COPD includes those with a diagnosis of chronic bronchitis or emphysema.

Participant characteristics varied by smoking status (Table 1) and sex (Table 2). Never-smokers were younger at lung cancer diagnosis (median 56.5 y, interquartile range: 46.0–65.0 y) with a wider spread of educational attainment. Distinctions between ever and never-smokers seemed to be driven by sex-specific differences. Just over one-in-five men (n = 87; 22.0%) and approximately three-quarters (n = 56; 74.0%) of women diagnosed with lung cancer were never-smokers. Most women in this cohort were either illiterate (n = 41, 53.3%) or educated to no more than the primary level (n = 18, 23.4%), whilst a majority were not working (n = 66, 85.7%) and had exposure to biomass pollution (n = 53, 68.8%). A greater proportion of women were diagnosed at stage 4 compared with men (80.3% versus 61.6%) (Supplementary Table 2).

**Table 2:** Descriptive Characteristics by Sex

Variable	Men (n = 395)	Women (n = 77)
Age (mean, SD) y	59.7 (10.6)	59.3 (10.6)
BMI (mean, SD) kg/m^2^	21.5 (3.8)	23.1 (4.4)
Education (n, %)		
Illiterate	98 (24.87%)	41 (53.25%)
Primary	113 (28.68%)	18 (23.38%)
Secondary (Matric)	80 (20.30%)	8 (10.39%)
Higher secondary (intermediate)	51 (12.94%)	1 (1.30%)
Graduate	38 (9.64%)	9 (11.69%)
Postgraduate	14 (3.55%)	0 (0.00%)
Occupation (n, %)		
Farming	93 (23.54%)	4 (5.19%)
Laborer/skilled worker/sales worker	176 (44.56%)	2 (2.60%)
White collar/office job	70 (17.72%)	5 (6.49%)
Unemployed	2 (0.51%)	0 (0.00%)
Retired/housewife	50 (12.66%)	66 (85.71%)
Other	4 (1.01%)	0 (0.00%)
Biomass exposure (n, %)	85 (21.52%)	53 (68.83%)
Asbestos exposure (n, %)	54 (13.67%)	1 (1.30%)
COPD^a^ (n, %)	82 (20.76%)	5 (6.49%)
Tuberculosis (n, %)	72 (18.23%)	11 (14.29%)
Smoking status (n, %)		
Never	87 (22.03%)	57 (74.03%)
Former	205 (51.90%)	18 (23.38%)
Current	103 (26.08%)	2 (2.60%)

Note: All data were complete except for education and asbestos exposure, in which one individual had no data.

BMI, body mass index; COPD, chronic obstructive pulmonary disease.

a COPD includes those with a diagnosis of chronic bronchitis or emphysema.

**Figure 1:**
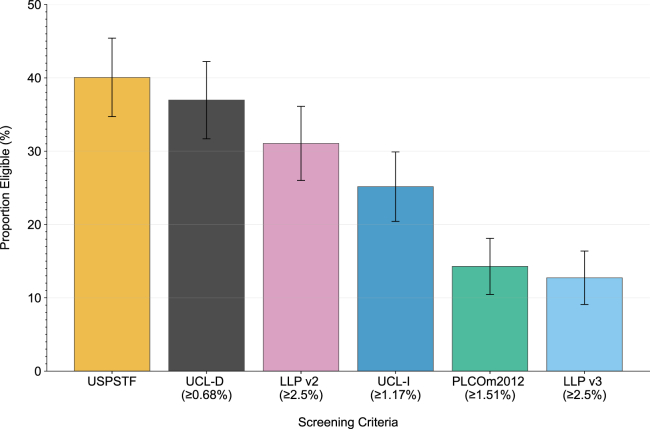
The percentage of ever-smokers who would have met the criteria for screening at the time of their lung cancer diagnosis. The USPSTF 2021 criteria includes those aged between 50 and 80 years with a 20 pack-year smoking history and, if a former smoker, have quit within the past 15 years. UCL-D estimates the absolute 5-year risk of death from cancer; all other risk models here estimate either the 5-year (LLP and UCL-I) or 6-year (PLCOm2012) absolute risk of cancer diagnosis. The percentages under each risk model reflect the threshold used in these analyses. LLP, Liverpool Lung Project; PLCOm2012, Prostate, Lung, Colorectal, and Ovarian Cancer Screening Trial modified 2012; USPSTF, U.S. Preventive Services Task Force.

We estimated the proportion of individuals who would have been theoretically eligible for lung cancer screening using criteria from the United States and the United Kingdom. Using the USPSTF criteria, 129 (40.1%) of the 322 ever-smokers in the cohort would have been eligible for lung cancer screening at the time of their diagnosis. An equivalent number of ever-smokers would have met a UCL-D threshold of a 5-year risk of death from lung cancer of 0.68% (n = 119, 37.0%), but only one-third, or fewer, of ever-smokers would have been eligible for lung cancer screening using the LLP, UCL-I, or PLCOm2012 models (Fig. 1, Supplementary Table 3). Existing risk models preferentially selected individuals who are older and have greater smoking histories (Supplementary Table 3). In our cohort, 68 individuals (21.1%) who had ever smoked were younger than 55 years at diagnosis and so would never have been considered for risk assessment under the existing U.K. protocol.^14^

## Discussion

Lung cancer screening is effective in reducing mortality among high-risk cohorts.^2^^,^^3^ In the United States, high-risk is determined on the basis of a combination of age and smoking history, whereas the United Kingdom uses risk models in conjunction with evidence-based risk thresholds. Among ever-smokers, fewer than 40% would have been eligible for screening using the risk models assessed. By contrast, in a U.S. case series, approximately 47% of ever-smokers with lung cancer would have met USPSTF-2021 criteria.^15^ This could reflect poor model performance in terms of discrimination or calibration in an Indian setting, inappropriate risk thresholds, and the fact that bidis—which may have a stronger link with lung cancer than cigarettes—are the dominant form of smoking.^16^ Another possible contributor is that risk models are known to preferentially select those at older ages with heavier smoking histories, which may not be appropriate in a context in which the average age at diagnosis is a decade or more lower than in the United States or United Kingdom, where these risk models were developed. Furthermore, nearly half (46.0%) of our cohort—rising to 76.6% of the women with lung cancer—would not have met eligibility criteria for risk assessment in the United Kingdom as they were either less than 55 years old or had never smoked. This suggests there are fundamental barriers to adapting existing eligibility criteria for screening either through recalibrating models or adjusting the risk thresholds.

In this cohort, 97% of patients were diagnosed with advanced cancer (stage III or IV), in keeping with other epidemiologic cohorts from India.^7^ For comparison, across five high-income countries between 2010 and 2014, approximately 75% of patients with lung cancer were diagnosed at an advanced stage.^17^ In tandem with other public health measures to tackle the causes of lung cancer, screening may require consideration to improve outcomes. It is increasingly recognized that one size may not fit all in designing lung cancer screening programs, particularly for implementation across Asia,^18^ and that there are unique challenges in establishing imaging-based screening programs because of the shortage of radiologists in India.^19^ There is, thus, a need to recalibrate existing models, develop India-specific models, or modify risk factor criteria analogous to the USPSTF to take account of region-specific factors. Any such approach will require the development of large prospective cohorts that represent the diversity of the Indian population, record relevant data, and have long-term follow-up for mortality. Furthermore, to effectively reduce the burden of lung cancer, screening research should be undertaken in conjunction with efforts to address tobacco control, biomass, and other air pollution.

This work has limitations. As a case series, we have had to apply risk models designed to predict future incidence or mortality of lung cancer among those already diagnosed with lung cancer. Correspondingly, the individual-level predictions will likely overestimate the risk that an individual would have had several years before their diagnosis. In addition, the available data do not allow us to quantify the performance of these models. However, we are not aware of any prospective cohorts from India with both relevant risk factor data and long-term follow-up to enable such an analysis. Furthermore, our case series reflects a convenience sample, reflecting an estimated 85% of all patients under treatment or active follow-up during the time-period at a single urban tertiary center, such that our findings may be subject to selection bias and not generalize more broadly.

In conclusion, existing criteria for lung cancer screening perform poorly in the Indian setting and are unlikely to be directly translatable to this population. Consequently, there is a need to modify these criteria on the basis of local characteristics, revalidate them in prospective cohorts, and consider randomized controlled trials of screening within an Indian context before widespread implementation.

## CRediT Authorship Contribution Statement

**Thomas Callender:** Conceptualization, Formal analysis, Writing - original draft, Writing - review and editing, Software, Visualization.

**Amyn Bhamani:** Writing - review and editing.

**Sneha Verma:** Data acquisition.

**Ayush Goel**: Data acquisition, Investigation.

**Tejas Suri**: Data acquisition, Investigation.

**John R Hurst:** Funding acquisition, Writing - review and editing.

**Neal Navani:** Supervision, Funding acquisition, Conceptualization, Writing - review and editing.

**Anant Mohan:** Funding acquisition, Conceptualization, Writing - review and editing, Data acquisition, Investigation, Ethical approval.

## Disclosure

Dr. Callender developed the lung cancer discrimination (UCL-D) and lung cancer incidence (UCL-I) models. The intellectual property (IP) for these models is owned by Mortimer Health Ltd., of which he is a founder and shareholder. Professor Navani is supported by a Medical Research Council Clinical Academic Research Partnership (MR/T02481X/1); has received grant funding from National Institute of Health Research, Cancer Research UK, EU-Horizon, Ruth Strauss Foundation, Engineering & Physical Sciences Research Council, and The Small Business Research Initiative in Healthcare; and reports receiving honoraria for nonpromotional educational talks, conference attendance, or advisory boards from Amgen, Astra Zeneca, AXANA, BeiGene, Boehringer Ingelheim, Bristol Myers Squibb, EQRx, Fujifilm, Guardant Health, Intuitive, Janssen, Lilly, Merck Sharp & Dohme, Olympus, Roche, and Sanofi. The remaining authors declare no conflict of interest.

## Footnotes

Note: To access the supplementary material accompanying this article, visit the online version of the *JTO Clinical and Research Reports* at www.jtocrr.org and at [https://doi.org/10.1016/j.jtocrr.2026.100987].
