# Supplementary material for: Brief Report: Translating Selection Criteria for Lung Cancer Screening to an Indian Context—An Analysis From a Tertiary Health Centre
Source: JTO Clin Res Rep. 2026 Mar 16;7(8):100987. doi: 10.1016/j.jtocrr.2026.100987 (PMC13351131; doi:10.1016/j.jtocrr.2026.100987)
Supplement: Appendix Table [file mmc1.docx]

**Appendix: Brief Report: Translating selection criteria for lung cancer screening to an Indian context – an analysis from a tertiary health centre**

Thomas Callender^1^, Amyn Bhamani^2^, Sneha Verma^3^, Ayush Goel^3^, Tejas Suri^3^, John R Hurst^4^, Neal Navani^2,5*^, Anant Mohan^3*^

**Author affiliations**

^1^ Department of Public Health and Primary Care, University of Cambridge, UK

^2^ Lungs for Living Research Centre, UCL Respiratory, University College London, UK

^3^ Department of Pulmonary, Critical Care and Sleep Medicine, All India Institute of Medical Sciences, New Delhi, India

^4^ UCL Respiratory, University College London, UK

^5^ University College London Hospitals NHS Foundation Trust, London, UK

| **Appendix Table 1:** Characteristics of lung cancers by smoking status | | | | | | | |
| --- | --- | --- | --- | --- | --- | --- | --- |
|  | | **All n=472** | | **Current smokers n=105** | **Former smokers n=223** | | **Never smokers n=144** |
| Histology (n, %) |  | |  | | |  | |
| Adenocarcinoma | | 184 (39.07%) | | 31 (29.52%) | 62 (27.93%) | | 91 (63.19%) |
| NSCLC (unspecified) | | 94 (19.96%) | | 21 (20.00%) | 43 (19.37%) | | 30 (20.83%) |
| Other | | 20 (4.25%) | | 7 (6.67%) | 9 (4.05%) | | 4 (2.78%) |
| Poorly differentiated carcinoma | | 8 (1.70%) | | 2 (1.90%) | 6 (2.70%) | | 0 (0.0%) |
| Small cell carcinoma | | 65 (13.80%) | | 21 (20.00%) | 37 (16.67%) | | 7 (4.86%) |
| Squamous cell carcinoma | | 100 (21.23%) | | 23 (21.90%) | 65 (29.28%) | | 12 (8.33%) |
| *Missing (n, %)* | | 1 (0.2%) | | 0 (0.0%) | 1 (0.4%) | | 0 (0.0%) |
| Stage (n, %) |  | |  | | |  | |
| 1 | | 3 (0.64%) | | 1 (0.95%) | 1 (0.45%) | | 1 (0.70%) |
| 2 | | 11 (2.36%) | | 2 (1.90%) | 8 (3.64%) | | 1 (0.70%) |
| 3 | | 151 (32.33%) | | 51 (48.57%) | 78 (35.45%) | | 22 (15.49%) |
| 4 | | 302 (64.67%) | | 51 (48.57%) | 133 (60.45%) | | 118 (83.10%) |
| *Missing (n, %)* | | 5 (1.1%) | | 0 (0.0%) | 3 (1.3%) | | 2 (1.4%) |
| Abbreviations: NSCLC, non-small cell lung cancer | | | | | | | |

| **Appendix Table 2:** Characteristics of lung cancers by sex | | |
| --- | --- | --- |
|  | **Men n=395** | **Women n=77** |
| Histology (n, %) | |  |
| Adenocarcinoma | 134 (34.01%) | 50 (64.94%) |
| NSCLC (unspecified) | 79 (20.05%) | 15 (19.48%) |
| Other | 18 (4.57%) | 2 (2.60%) |
| Poorly differentiated carcinoma | 7 (1.78%) | 1 (1.30%) |
| Small cell carcinoma | 59 (14.97%) | 6 (7.79%) |
| Squamous cell carcinoma | 97 (24.62%) | 3 (3.90%) |
| *Missing (n, %)* | 1 (0.3%) | 0 (0.0%) |
| Stage (n, %) | |  |
| 1 | 3 (0.77%) | 0 (0.00%) |
| 2 | 9 (2.30%) | 2 (2.63%) |
| 3 | 138 (35.29%) | 13 (17.11%) |
| 4 | 241 (61.64%) | 61 (80.26%) |
| *Missing (n, %)* | 4 (1.0%) | 1 (1.3%) |

| **Appendix Table 3:** Characteristics of ever-smokers eligible for lung cancer screening by risk model | | | | | | | | | | | | | |
| --- | --- | --- | --- | --- | --- | --- | --- | --- | --- | --- | --- | --- | --- |
|  | | **All ever-smokers n=322** | | **USPSTF eligible ever-smokers n=129** | | **UCL-D eligible ever-smokers n=119** | **UCL-I eligible ever-smokers n=81** | | **PLCOm2012 eligible ever-smokers n=46** | | **LLPv2 eligible ever-smokers n=100** | | **LLPv3 eligible ever-smokers n=41** |
| Age (mean, SD) years | | 61.8 (8.6) | | 63.8 (6.4) | | 66.7 (6.3) | 67.4 (5.7) | | 68.7 (4.8) | | 67.9 (5.8) | | 71.2 (4.6) |
| *Missing (n, %)* | | 0 (0.0%) | | 0 (0.0%) | | 0 (0.0%) | 0 (0.0%) | | 0 (0.0%) | | 0 (0.0%) | | 0 (0.0%) |
| Sex - Female (n, %) | | 20 (6.21%) | | 6 (4.65%) | | 7 (5.88%) | 3 (3.70%) | | 4 (8.70%) | | 2 (2.00%) | | 2 (4.88%) |
| Missing (n, %) | | 0 (0.0%) | | 0 (0.0%) | | 0 (0.0%) | 0 (0.0%) | | 0 (0.0%) | | 0 (0.0%) | | 0 (0.0%) |
| Education (n, %) |  | |  | |  | | |  | |  | |  | |
| Illiterate | | 85 (26.48%) | | 32 (25.00%) | | 30 (25.21%) | 19 (23.46%) | | 12 (26.09%) | | 25 (25.00%) | | 7 (17.07%) |
| Primary | | 107 (33.33%) | | 53 (41.41%) | | 48 (40.34%) | 33 (40.74%) | | 21 (45.65%) | | 37 (37.00%) | | 14 (34.15%) |
| Secondary (Matric) | | 59 (18.38%) | | 19 (14.84%) | | 16 (13.45%) | 11 (13.58%) | | 7 (15.22%) | | 13 (13.00%) | | 7 (17.07%) |
| Higher secondary (intermediate) | | 40 (12.46%) | | 14 (10.94%) | | 12 (10.08%) | 8 (9.88%) | | 4 (8.70%) | | 12 (12.00%) | | 8 (19.51%) |
| Graduate | | 24 (7.48%) | | 8 (6.25%) | | 10 (8.40%) | 7 (8.64%) | | 2 (4.35%) | | 10 (10.00%) | | 2 (4.88%) |
| Post-graduate | | 6 (1.87%) | | 2 (1.56%) | | 3 (2.52%) | 3 (3.70%) | | 0 (0.00%) | | 3 (3.00%) | | 3 (7.32%) |
| *Missing (n, %)* | | 1 (0.3%) | | 1 (0.8%) | | 0 (0.0%) | 0 (0.0%) | | 0 (0.0%) | | 0 (0.0%) | | 0 (0.0%) |
| Occupation (n, %) |  | |  | |  | | |  | |  | |  | |
| Farming | | 86 (26.71%) | | 42 (32.56%) | | 38 (31.93%) | 28 (34.57%) | | 17 (36.96%) | | 32 (32.00%) | | 17 (41.46%) |
| Labourer/skilled worker/sales worker | | 149 (46.27%) | | 62 (48.06%) | | 53 (44.54%) | 33 (40.74%) | | 21 (45.65%) | | 42 (42.00%) | | 12 (29.27%) |
| White collar/office job | | 49 (15.22%) | | 14 (10.85%) | | 14 (11.76%) | 12 (14.81%) | | 4 (8.70%) | | 17 (17.00%) | | 4 (9.76%) |
| Unemployed | | 2 (0.62%) | | 1 (0.78%) | | 2 (1.68%) | 2 (2.47%) | | 1 (2.17%) | | 2 (2.00%) | | 1 (2.44%) |
| Retired/housewife | | 33 (10.25%) | | 9 (6.98%) | | 11 (9.24%) | 6 (7.41%) | | 3 (6.52%) | | 7 (7.00%) | | 7 (17.07%) |
| Other | | 3 (0.93%) | | 1 (0.78%) | | 1 (0.84%) | 0 (0.00%) | | 0 (0.00%) | | 0 (0.00%) | | 0 (0.00%) |
| *Missing (n, %)* | | 0 (0.0%) | | 0 (0.0%) | | 0 (0.0%) | 0 (0.0%) | | 0 (0.0%) | | 0 (0.0%) | | 0 (0.0%) |
| Smoking Type (n, %) |  | |  | |  | | |  | |  | |  | |
| Bidi | | 266 (82.61%) | | 108 (83.72%) | | 94 (78.99%) | 63 (77.78%) | | 36 (78.26%) | | 79 (79.00%) | | 31 (75.61%) |
| Cigarettes | | 26 (8.07%) | | 8 (6.20%) | | 9 (7.56%) | 5 (6.17%) | | 4 (8.70%) | | 8 (8.00%) | | 4 (9.76%) |
| Both | | 30 (9.32%) | | 13 (10.08%) | | 16 (13.45%) | 13 (16.05%) | | 6 (13.04%) | | 13 (13.00%) | | 6 (14.63%) |
| *Missing (n, %)* | | 0 (0.0%) | | 0 (0.0%) | | 0 (0.0%) | 0 (0.0%) | | 0 (0.0%) | | 0 (0.0%) | | 0 (0.0%) |
| Smoking status (n, %) |  | |  | |  | | |  | |  | |  | |
| Former | | 218 (67.70%) | | 80 (62.02%) | | 74 (62.18%) | 52 (64.20%) | | 46 (100.00%) | | 65 (65.00%) | | 28 (68.29%) |
| Current | | 104 (32.30%) | | 49 (37.98%) | | 45 (37.82%) | 29 (35.80%) | | 0 (0.00%) | | 35 (35.00%) | | 13 (31.71%) |
| *Missing (n, %)* | | 0 (0.0%) | | 0 (0.0%) | | 0 (0.0%) | 0 (0.0%) | | 0 (0.0%) | | 0 (0.0%) | | 0 (0.0%) |
| Years smoked (mean, SD) | | 29.7 (13.6) | | 38.8 (10.2) | | 43.2 (8.1) | 46.5 (6.9) | | 42.0 (10.3) | | 44.1 (9.1) | | 49.3 (7.5) |
| *Missing (n, %)* | | 2 (0.6%) | | 0 (0.0%) | | 0 (0.0%) | 0 (0.0%) | | 0 (0.0%) | | 0 (0.0%) | | 0 (0.0%) |
| Cigarettes per day (mean, SD) | | 14.8 (11.4) | | 22.8 (13.1) | | 20.0 (14.3) | 22.3 (15.6) | | 27.2 (17.8) | | 18.5 (12.8) | | 18.3 (12.8) |
| *Missing (n, %)* | | 4 (1.2%) | | 0 (0.0%) | | 0 (0.0%) | 0 (0.0%) | | 0 (0.0%) | | 0 (0.0%) | | 0 (0.0%) |
| Pack-years (mean, SD) | | 24.1 (25.2) | | 44.1 (29.0) | | 42.9 (31.2) | 50.6 (33.9) | | 56.3 (37.0) | | 41.2 (31.1) | | 45.2 (33.0) |
| *Missing (n, %)* | | 4 (1.2%) | | 0 (0.0%) | | 0 (0.0%) | 0 (0.0%) | | 0 (0.0%) | | 0 (0.0%) | | 0 (0.0%) |
| Abbreviations: SD, standard deviation; USPSTF, US Preventive Services Task Force; LLP, Liverpool Lung Project. | | | | | | | | | | | | | |
